# Enhanced PRL-1 expression in placenta-derived mesenchymal stem cells accelerates hepatic function via mitochondrial dynamics in a cirrhotic rat model

**DOI:** 10.1186/s13287-020-02029-3

**Published:** 2020-11-27

**Authors:** Jae Yeon Kim, Jong Ho Choi, Ji Hye Jun, Sohae Park, Jieun Jung, Si Hyun Bae, Gi Jin Kim

**Affiliations:** 1 Department of Biomedical Science, CHA University, Seongnam, 13488 Republic of Korea; 2 Department of Oral Pathology, College of Dentistry, Gangneung-Wonju National University, Gangneung, 25457 Republic of Korea; 3 Department of Internal Medicine, Catholic University Medical College, Seoul, 06591 Republic of Korea

**Keywords:** Chronic liver fibrosis, Mitochondria, Phosphatase of regenerating liver-1, Placenta-derived mesenchymal stem cells

## Abstract

**Background:**

Placenta-derived mesenchymal stem cells (PD-MSCs) have been highlighted as an alternative cell therapy agent that has become a next-generation stem cell treatment. Phosphatase of regenerating liver-1 (PRL-1), an immediate early gene, plays a critical role during liver regeneration. Here, we generated enhanced PRL-1 in PD-MSCs (PD-MSCs^PRL-1^, PRL-1+) using lentiviral and nonviral gene delivery systems and investigated mitochondrial functions by PD-MSC^PRL-1^ transplantation for hepatic functions in a rat bile duct ligation (BDL) model.

**Methods:**

PD-MSCs^PRL-1^ were generated by lentiviral and nonviral AMAXA gene delivery systems and analyzed for their characteristics and mitochondrial metabolic functions. Liver cirrhosis was induced in Sprague-Dawley (SD) rats using common BDL for 10 days. PKH67+ naïve and PD-MSCs^PRL-1^ using a nonviral sysyem (2 × 10^6^ cells/animal) were intravenously administered into cirrhotic rats. The animals were sacrificed at 1, 2, 3, and 5 weeks after transplantation and engraftment of stem cells, and histopathological analysis and hepatic mitochondrial functions were performed.

**Results:**

PD-MSCs^PRL-1^ were successfully generated using lentiviral and nonviral AMAXA systems and maintained characteristics similar to those of naïve cells. Compared with naïve cells, PD-MSCs^PRL-1^ improved respirational metabolic states of mitochondria. In particular, mitochondria in PD-MSCs^PRL-1^ generated by the nonviral AMAXA system showed a significant increase in the respirational metabolic state, including ATP production and mitochondrial biogenesis (*p < 0.05). Furthermore, transplantation of PD-MSCs^PRL-1^ using a nonviral AMAXA system promoted engraftment into injured target liver tissues of a rat BDL cirrhotic model and enhanced the metabolism of mitochondria via increased mtDNA and ATP production, thereby improving therapeutic efficacy.

**Conclusions:**

Our findings will further our understanding of the therapeutic mechanism of enhanced MSCs and provide useful data for the development of next-generation MSC-based cell therapy and therapeutic strategies for regenerative medicine in liver disease.

## Introduction

Regenerative medicine using stem cells is a new promising field for treating damaged organs and various degenerative diseases, such as difficult-to-treat hepatic failure disease. Various stem cells have the capacity to ameliorate liver damage in animal models of hepatic failure. Chronic liver disease is characterized by permanent changes to the liver and is associated with poor outcomes and high mortality. Although the most effective therapy for acute hepatic failure and advanced cirrhosis is liver transplantation, this procedure has several limitations, including surgical complications, immunological suppression, and high medical cost [1].

The function of mitochondria in the liver following hepatic failure is important for liver regeneration [2]. Hepatic diseases cause defects in mitochondrial structure and function, as the mitochondria in the hepatocytes of patients with nonalcoholic steatohepatitis are swollen and exhibit abnormal morphology with a loss of cristae; additionally, in these patients, the activities of mitochondrial respiratory complexes are impaired, and mitochondrial oxidative stress is increased [3, 4]. Moreover, the dynamics of mitochondrial biogenesis are essential to liver regeneration [5]. Augmentation of liver regeneration knockdown inhibits mitochondrial transcription factor A (mtTFA) and peroxisome proliferator-activated receptor gamma coactivator-1 alpha (PGC-1α), resulting in impaired mitochondrial biogenesis [6].

In hepatic failure diseases, hepatic restoration by bone marrow-derived mesenchymal stem cell (BM-MSC) transplantation is associated with the mitochondria-dependent pathway [7]. The therapeutic action of MSCs in liver disease has been postulated to include differentiation into hepatocytes or fusion with endogenous hepatocytes [8]. MSCs reduce liver injury by ameliorating oxidative stress through the release of antioxidants and antifibrotic effects [9]. Recently, autologous MSC transplantation into patients with hepatic diseases was attempted in clinical trials [10, 11]. Clinical trials have shown that BM-MSC transfusion can improve hepatic function [12]. Although hepatic function tests were improved in several patients, the trial triggered side effects [13]. In particular, isolation of autologous MSCs in patients has limitations, including pain and a lack of fresh MSCs harvested from patients.

Placenta-derived mesenchymal stem cells (PD-MSCs) have very strong immune-modulatory privileges [14]. Recently, we reported that increased autophagy induced by PD-MSC transplantation promotes hepatic regeneration in carbon tetrachloride (CCl_4_)-induced hepatic cirrhotic rats [15]. In addition, epigenetic alterations induced by PD-MSCs through the interleukin-6 (IL-6)/signal transducer and activator of transcription 3 (STAT3) signaling pathway improve liver regeneration [16]. Moreover, human placental MSC administration significantly decreased transforming growth factor-β1 and alpha smooth muscle actin expression and enhanced liver function in CCl_4_-induced fibrotic rats [17].

Phosphatase of regenerating liver-1 (PRL-1), a protein tyrosine phosphatase, was originally identified as an immediate early gene in liver regeneration [18]. The function of PRL-1 is to promote cell migration and invasion by stimulating matrix metalloproteinase (MMP)-2 and MMP-9 expression via the Src kinase and ERK1/2 pathways; additionally, PRL-1 binds to p115 Rho GTPase-activating protein [19, 20]. Ectopic expression of PRL-1 increases Rho levels, which have a critical role in actin filament assembly and focal adherin stabilization [21]. PRL-1 expression protects cells from apoptosis and is essential for the normal timing of cell cycle progression during liver regeneration [22]. Moreover, PRL-1 regulates hematopoietic stem cell behavior [23, 24].

However, the effect of enhanced PRL-1 expression in PD-MSCs (PD-MSCs^PRL-1^, PRL-1+) on mitochondrial function properties in hepatic cirrhotic liver remains unclear. Therefore, the objective of this study is to generate stable PD-MSCs^PRL-1^ using lentiviral and nonviral systems, compare their characteristics, and demonstrate their therapeutic effects on liver function through the mitochondrial metabolic pathway in a rat cirrhosis model established by bile duct ligation (BDL).

## Materials and methods

### Cell culture

Placentas at term (≥ 37 gestational weeks) were assembled from women without medical, obstetrical, and surgical complications. The collection of samples and their use for research purposes were approved by the Institutional Review Board (IRB) of CHA General Hospital, Seoul, Korea (IRB 07-18). All participating women provided written, informed consent. PD-MSCs were harvested as previously described [25]. PD-MSCs were maintained in alpha-modified minimal essential medium (α-MEM; HyClone, Logan, UT, USA) supplemented with 10% fetal bovine serum (FBS; Gibco, Carlsbad, CA, USA), 1% penicillin/streptomycin (P/S; Gibco), 25 ng/ml human fibroblast growth factor-4 (hFGF-4) (PeproTech, Rocky Hill, NJ, USA), and 1 μg/ml heparin (Sigma-Aldrich, St. Louis, MO, USA). Primary hepatocytes from 7-week-old male Sprague-Dawley (SD) rats (Orient Bio Inc., Seongnam, Korea) were isolated using a two-step collagenase perfusion process and were cultured in William’s E medium (Sigma-Aldrich) supplemented with 10% FBS (Gibco), 1% P/S (Gibco), and 4 mM L-glutamine (Gibco). To induce cholestatic injury in primary hepatocytes, 100 μM lithocholic acid (LCA) was applied for 12 h. Naïve and PD-MSCs^PRL-1^ (1 × 10^5^ cells) were seeded onto Transwell inserts (8-μm pore size; Corning, NY, USA). Cells were maintained at 37 °C in a humidified atmosphere containing 5% CO_2_.

### Gene transfections

The PRL-1 plasmid (human protein tyrosine phosphatase type 4 A, member 1; PTP4A1) was purchased (Origene Inc., Rockville, MD, USA) and used to induce the overexpression of the PRL-1 gene. The CMV6-AC vector containing PRL-1, the GFP reporter gene, CMV promoters, and the antibiotic neomycin was digested with Sgf1 and Mlu1 restriction enzymes. The PRL-1 lentiviral plasmid was purchased from SeouLin Bioscience (Seongnam, Korea). The pLenti-RSV-EF1α vector including PRL-1 was constructed with a C-terminal GFP as well as the antibiotic puromycin. The AMAXA pCMV-GFP vector was obtained from Lonza (Basel, Switzerland). The resulting plasmid was confirmed by DNA sequencing. Naïve PD-MSCs (6 × 10^4^ cells/cm^2^) were harvested and transfected by using an AMAXA system with a Human MSC Nucleofector Kit (Lonza) and lentiviral vector (SeouLin Bioscience). After transfections by each system, cells generated using AMAXA were selected by using 1.5 mg/ml neomycin. In addition, cells generated using lentiviral vector were selected by using 2 μg/ml puromycin for 7 days. We changed the medium every other day and observed changes in cell morphology. Cells were maintained at 37 °C in a humidified atmosphere containing 5% CO_2_.

### Animal models and transplantation of MSCs

Seven-week-old male SD rats (Orient Bio Inc.) were housed under specific pathogen-free conditions. Liver cirrhosis was induced by common BDL as previously described [26]. Naïve cells (TTx naïve; *n* = 20) and PD-MSCs^PRL-1^ (TTx PRL-1+; *n* = 20) (2 × 10^6^ cells, 9–10 passages) were stained with the PKH67 Fluorescent Cell Linker Kit (Sigma-Aldrich) and transplanted through the tail vein. Nontransplanted (NTx; *n* = 20) rats were maintained as well as sham controls (Con; *n* = 5). After 1, 2, 3, and 5 weeks, the rats were sacrificed, and their liver tissues and blood were harvested. Alanine aminotransferase (ALT), aspartate aminotransferase (AST), total bilirubin (TBIL), and albumin levels were measured using separated serum from the blood (Southeast Medi-Chem Institute, Busan, Korea). In all animal experimental processes, protocols were approved by the Institutional Animal Care Use Committee (IACUC) of CHA University, Seongnam, Korea (IACUC-180023).

### Histopathological and immunofluorescence analysis

To confirm the induction of liver cirrhosis with BDL and engraftment into target injured tissue for histological examination, liver specimens for each group (*n* = 5) were collected and fixed in 10% neutral buffered formalin (NBF). Samples were embedded in paraffin and OCT compound and processed as 5-μm-thick sections for hematoxylin and eosin (H&E), Sirius red, Masson’s trichrome, and PKH67+ (green) staining. 4′,6-Diamidino-2-phenylindole (DAPI) (Invitrogen, Carlsbad, CA, USA) was used as a counterstain for immunofluorescence. Morphometric images of whole sections in the liver were captured using a digital slide scanner (3DHISTECH Ltd., Budapest, Hungary).

### Reverse transcription and quantitative real-time polymerase chain reaction (PCR) analysis

Total RNA was extracted from samples with TRIzol LS Reagent (Invitrogen). cDNA was synthesized by reverse transcription from total RNA (500 ng) using SuperScript III reverse transcriptase (Invitrogen) according to the manufacturer’s instructions. To analyze stemness and hepatogenic differentiation markers of naïve and PD-MSCs^PRL-1^, PCR amplification was performed with specific primers (Table 1, designed by BIONEER, Daejeon, Korea). β-actin was used as an internal control.

**Table 1:** Primer sequences using reverse transcription polymerase chain reaction

Genes		Primer sequences	Tm
Oct4	Forward	5′-AGTGAGAGGCAACCTGGAGA-3′	52
	Reverse	5′-GTGAAGTGAGGGCTCCCATA-3′	
Nanog	Forward	5′-TTCTTGACTGGGACCTTGTC-3′	52
	Reverse	5′-GCTTGCCTTGCTTTGAAGCA-3′	
Sox2	Forward	5′-GGGCAGCGTGTACTTATCCT-3′	52
	Reverse	5′-AGAACCCCAAGATGCACAAC-3′	
HLA-G	Forward	5′-GCGGCTACTACAACCAGAGC-3′	58
	Reverse	5′-GCACATGGCACGTGTATCTC-3′	
TERT	Forward	5′-GAGCTGACGTGGAAGATGAG-3′	55
	Reverse	5′-CTTCAAGTGCTGTCTGATTCCAATG-3′	
Albumin	Forward	5′-TGAGTTTGCAGAAGTTTCCA-3′	60
	Reverse	5′-CCTTTGCCTCAGCATAGTTT-3′	
TAT	Forward	5′-AACGATGTGGAGTTCACGG-3′	55
	Reverse	5′-GACACATCCTCAGGAGAATGG-3′	
HNF1A	Forward	5′-TACACCACTCTGGCAGCCACACT-3′	60
	Reverse	5′-CGGTGGGTACATACCTGACAGAAC-3′	
CYP2B6	Forward	5′-CTTGACCTGCTGCTTCTTCC-3′	55
	Reverse	5′-TGCTTCCCGCCTCAGATTTCTC-3′	
CYP3A4	Forward	5′-ATCATTGCTGTCTCCAACCTTCAC-3′	60
	Reverse	5′-TGCTTCCCGCCTCAGATTTCTC-3′	
β-actin	Forward	5′-TCCTTCTGCATCCTGTCAGCA-3′	58
	Reverse	5′-CAGGAGATGGCCACTGCCGCA-3′	

To assess the differentiation, migration, mitochondrial biogenesis, and albumin expression of samples, qRT-PCR was performed with human and rat primers (Tables 2 and 3, designed by BIONEER) and SYBR Green Master Mix (Roche, Basel, Switzerland) in a CFX Connect™ Real-Time System (Bio-Rad, Hercules, CA, USA). Target gene expression was normalized to GAPDH, and the 2−ΔΔCT method generated the relative values of mRNA expression. All reactions were performed in triplicate.

**Table 2:** Human primer sequences using quantitative real-time polymerase chain reaction

Genes		Primer sequences	Tm
OC	Forward	5′-CACTCCTCGCCCTATTGGC-3′	58
	Reverse	5′-CCCTCCTGCTTGGACACAAAG-3′	
COL1A1	Forward	5′-AGACATCCCACCAATCACCT-3′	60
	Reverse	5′-CGTCATCGCACAACACCT-3′	
Adipsin	Forward	5′-GGTCACCCAAGCAACAAAGT-3′	60
	Reverse	5′-CCTCCTGCGTTCAAGTCATC-3′	
PPAR-γ	Forward	5′-TTGACCCAGAAAGCGATTCC-3′	60
	Reverse	5′-AAAGTTGGTGGGCCAGAATG-3′	
PRL-1	Forward	5′-TACTGCTCCACCAAGAAGCC-3′	60
	Reverse	5′-AGGTTTACCCCATCCAGGTC-3′	
RhoA	Forward	5′-TGGAAAGCAGGTAGAGTTGG-3′	60
	Reverse	5′-GACTTCTGGGGTCCACTTTT-3′	
ROCK1	Forward	5′-GAAGAAAGAGAAGCTCGAGA-3′	60
	Reverse	5′-GATCTTGTAGCTCCCGCATCTGT-3′	
Alu	Forward	5′-GGAGGCTGAGGCAGGAGAA-3′	55
	Reverse	5′-CGGAGTCTCGCTCTGTCGCCCA-3′	
PGC-1α	Forward	5′-CAGCAAAAGCCACAAAGACG-3′	60
	Reverse	5′-GGGTCAGAGGAAGAGATAAAGTTG-3′	
NRF1	Forward	5′-GCTTCAGAATTGCCAACCAC-3′	60
	Reverse	5′-GTCATCTCACCTCCCTGTAAC-3′	
mtTFA	Forward	5′-GAACAACTACCCATATTTAAAGCTCA-3′	60
	Reverse	5′-GAATCAGGAAGTTCCCTCCA-3′	
mtDNA	Forward	5′-CCACTGTAAAGCTAACTTAGCATTAACC-3′	55
	Reverse	5′-GTGATGAGGAATAGTGTAAGGAGTATGG-3′	
nuclearDNA	Forward	5′-CCAGAAAATAAATCAGATGGTATGTAACA-3′	55
	Reverse	5′-TGGTTTAGGAGGGTTGCTTCC-3′	
GAPDH	Forward	5′-GCACCGTCAAGGCTGAGAAC-3′	60
	Reverse	5′-GTGGTGAAGACGCCAGTGGA-3′

**Table 3:** Rat primer sequences using quantitative real-time polymerase chain reaction

Genes		Primer sequences	Tm
PGC-1α	Forward	5′-GTGCAGCCAAGACTCTGTATGG-3′	60
	Reverse	5′-GTCCAGGTCATTCACATCAAGTTC-3′	
NRF1	Forward	5′-GCTGTCCCACTCGTGTCGTAT-3′	60
	Reverse	5′-GTTTGAGTCTAACCCATCTATCCG-3′	
mtTFA	Forward	5′-CGCCTAAAGAAGAAAGCACA-3′	60
	Reverse	5′-GCCCAACTTCAGCCATTT-3′	
mtD-Loop	Forward	5′-GGTTCTTACTTCAGGGCCATCA-3′	55
	Reverse	5′-GATTAGACCCGTTACCATCGAGAT-3′	
β-actin	Forward	5′-GGGATGTTTGCTCCAACCAA-3′	55
	Reverse	5′-GCGCTTTTGACTCAAGGATTTAA-3′	
GAPDH	Forward	5′-TCCCTCAAGATTGTCAGCAA-3′	60
	Reverse	5′-AGATCCACAACGGATACATT-3′	

### Western blotting

To assess the specific gene expression of PD-MSCs^PRL-1^ and cirrhotic liver tissues, samples were lysed in protein lysis buffer (Sigma-Aldrich). The protein lysates were loaded on sodium dodecyl sulfate polyacrylamide gels, and separated proteins were transferred to PVDF membranes. The following primary antibodies were used: anti-human PRL-1, anti-Oct4 (1:1000; all from Abcam, Cambridge, UK), anti-albumin, anti-HLA-G (4 h84) (1:1000; all from Novus Biologicals, Littleton, CO, USA), anti-ATP5B (1:200; Santa Cruz Biotechnology, Dallas, TX, USA), anti-ROCK1, anti-RhoA, anti-CDK4, anti-cyclin D1, and mitochondrial marker antibody sampler kit (1:1000; all from Cell Signaling Technology, Denvers, MA, USA). The loading control was anti-GAPDH (1:3000; AbFrontier, Seoul, Korea). The following secondary antibodies were used: anti-mouse IgG (1:5000; Bio-Rad, Hercules, CA, USA) and anti-rabbit IgG (1:10,000; Bio-Rad). The bands were detected using ECL reagent (Bio-Rad).

### Multilineage differentiation analysis

To induce adipogenic and osteogenic differentiation, PD-MSCs^PRL-1^ (5 passages) generated using lentiviral and AMAXA systems were plated (5 × 10^3^ cells/cm^2^) in each differentiation induction medium using a StemPro Adipogenesis and Osteogenesis differentiation kit (Gibco). Each medium was changed every other day. After approximately 21 days, cells were fixed with 4% paraformaldehyde and incubated for 1 h with Oil Red O (Sigma-Aldrich) for staining lipids and von Kossa with 5% silver nitrate (Sigma-Aldrich) to visualize the calcium deposits.

To induce hepatogenic differentiation, PD-MSCs^PRL-1^ (5 passages) were seeded (5 × 10^3^ cells/cm^2^) in low-glucose Dulbecco’s modified Eagle medium (DMEM; HyClone) supplemented with 40% MCDB 201 medium (Sigma-Aldrich), 2% FBS, and 1% P/S coated with 5 μg/ml type IV collagen. After 48 h, the growth medium was replaced with low-glucose DMEM supplemented with 20 ng/ml epidermal growth factor (EGF; Peprotech), 10 ng/ml basic fibroblast growth factor (bFGF; Peprotech), 10 ng/ml bone morphogenetic protein-4 (Peprotech), 40% MCDB 201 medium, and 1% P/S. Then, PD-MSCs^PRL-1^ were cultured for an additional 48 h. The cells were treated for another week in step-1 medium consisting of low-glucose DMEM supplemented with 20 ng/ml hepatocyte growth factor (Peprotech), 10 ng/ml bFGF, 40% MCDB 201 medium, 2% FBS, and 1% P/S to progress to the maturation step. Then, differentiation was induced by incubating PD-MSCs^PRL-1^ for another week with step-2 medium consisting of low-glucose DMEM supplemented with 20 ng/ml oncostatin M (Peprotech), 1 μM dexamethasone (Sigma-Aldrich), 1× ITS+ Premix (Sigma-Aldrich), 40% MCDB 201 medium, 2% FBS, and 1% P/S. During steps 1 and 2, the culture media were changed every other day. After 18 days, each plate of PD-MSCs^PRL-1^ was incubated for 1 h at 37 °C with 1 mg/ml indocyanine green (ICG) (Dong in Dang Pharm., Siheung, Korea) uptake as described previously [25].

### FACS analysis

PD-MSCs^PRL-1^ (5 passages) were stained with monoclonal antibodies specific for the following proteins to phenotype cell-surface antigens: CD34 (PE), CD90 (PE), HLA-ABC (FITC), HLA-DR (FITC) (BD Bioscience, San Diego, CA, USA), CD13 (PE) (BioLegend, San Diego, CA, USA), CD105 (PE) (R&D Systems, Minneapolis, MN, USA), and HLA-G (FITC) (Abcam). After staining, cells were washed in PBS and treated with appropriate isotype antibodies (BD Biosciences, San Jose, CA, USA). Cells were analyzed by a FACSCalibur flow cytometer (Becton Dickinson, Franklin Lakes, NJ, USA). For each sample, at least 10,000 events were acquired.

### Teratoma formation

To confirm teratoma formation by PD-MSCs^PRL-1^, 9-week-old male nonobese diabetic/severe combined immunodeficiency (NOD/SCID) mice (Laboratory Animal Research Center, Bungdang CHA Medical Center, Seongnam, Korea) were housed in an air-conditioned animal facility under pathogen-free conditions. A total of 5 × 10^5^ cells of each cell type (lenti and AMAXA) were transplanted into one testis (Tx; *n* = 2). The other testis was not injected (Con; *n* = 2). After the testes were maintained for 14 weeks, the mice were sacrificed, and the testes of all groups were collected. Each section of testis tissue was stained with H&E.

### Migration assay

Naïve and PD-MSCs^PRL-1^ (2 × 10^4^ cells) were seeded into the upper inserts of a Transwell chamber (8-μm pore size; Corning) with or without siRNA-PRL-1 after 24 h to analyze the migration ability of naïve and PD-MSCs^PRL-1^. A scrambled siRNA (Invitrogen) was used as a negative control (NC). Each cell type was fixed with 100% methanol for 10 min and then stained with Mayer’s hematoxylin (Dako, Santa Clara, CA, USA) for 5 min. The number of stained cells was randomly counted in eight nonoverlapping fields on the membranes at a magnification of × 200. The experiments were performed in triplicate.

### L-lactate production assay

To confirm the end product of glycolysis in naïve and PD-MSCs^PRL-1^, the lactate production rate was determined using a colorimetric L-lactate assay kit (Abcam). Cell lysates were deproteinized to eliminate endogenous LDH by a Deproteinizing Sample Preparation Kit-TCA (Abcam). Each sample was plated in a 96-well plate, and lactate reagent was added for 30 min. The absorbance was quantified using an Epoch microplate reader (BioTek, Winooski, VT, USA) at 450 nm. The lactate concentration was evaluated by the trend line equation. The experiments were conducted in triplicate.

### Measurement of ATP production

To confirm that mitochondria provide energy, ATP concentrations of homogenized liver tissue samples, PD-MSCs^PRL-1^, and primary hepatocyte lysate were measured by an ATP determination kit (Thermo Fisher Scientific, Waltham, MA, USA). According to the manufacturer’s instructions, ATP concentrations were assessed using a microplate reader (BioTek) at 570 nm. The experiments were performed in triplicate.

### Mitochondrial DNA (mtDNA) copy number assay

Genomic DNA (gDNA) was extracted from MSCs, primary hepatocytes, and homogenized BDL-injured rat liver to analyze mtDNA copy number. qRT-PCR amplification was conducted with specific primers (Table 2) containing 250 ng of gDNA, primers of nuclear DNA with FAM- and mtDNA with JOE-labeled quencher dye, and 1× TaqMan Universal Master Mix (Applied Biosystems, Foster City, CA, USA) according to the manufacturer’s instructions. Data were obtained in triplicate.

### XF Mito Stress assay

To further analyze mitochondrial metabolic functions in live PD-MSCs^PRL-1^, an XF24 Extracellular Flux Analyzer (Seahorse Bioscience, North Billerica, MA, USA) was used for real-time analysis of the oxygen consumption rate (OCR) and extracellular acidification rate (ECAR). Naïve and PD-MSCs^PRL-1^ were plated on XF24-well microplates (Seahorse Bioscience) (7 × 10^3^ cells/well). The cells were adjusted for equilibrium in XF buffer for 60 min for contemporaneous analysis of OCR and ECAR by repeated cycles of mixing (3 min), incubation (2 min), and measurement (3 min) periods in a non-CO_2_ incubator. Following basal respiration measurements, the cells were sequentially treated with 0.5 μM oligomycin, 0.5 μM carbonyl cyanide-*p*-trifluoromethoxyphenylhydrazone (FCCP), and 1 μM rotenone/antimycin A (AA) mixture. The changes in respiration were recorded. The Seahorse XF24 Analyzer program was set according to the manufacturer’s recommendation. OCR/ECAR was then normalized by total cell number.

### Statistical analysis

All data are expressed as the mean ± standard deviation (SD). Student’s *t* test was used for analysis, and *p* values less than 0.05 were considered statistically significant. All experiments were conducted in triplicate.

## Results

### Generation of stable PD-MSCs with PRL-1 using lentiviral and nonviral AMAXA systems

**Fig. 1:**
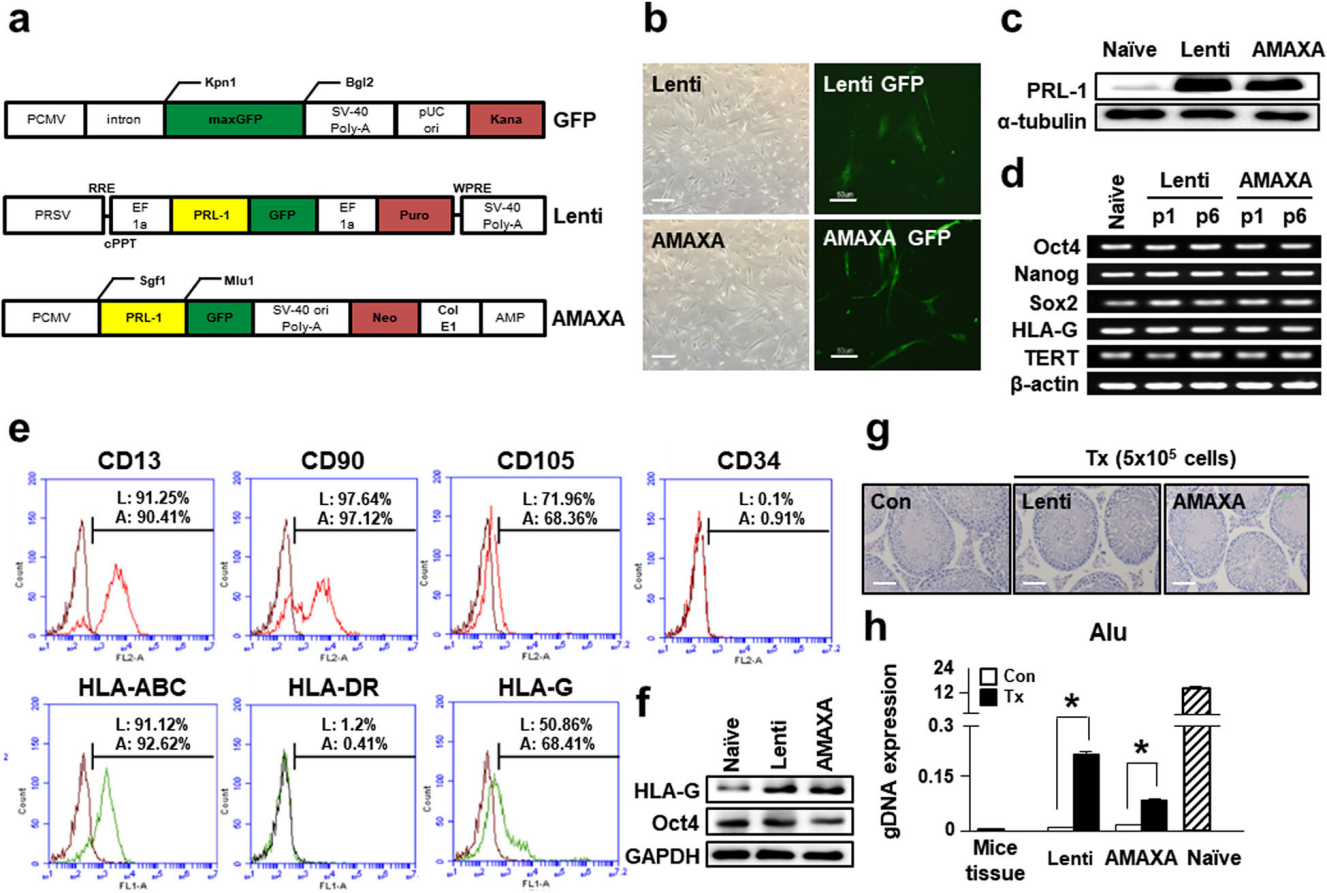
Generation of stable PD-MSCs with PRL-1 (PD-MSCs^PRL-1^, PRL-1+) using lentiviral and nonviral AMAXA systems. **a** GFP, lentiviral, and nonviral AMAXA plasmid vector map. **b** Morphology of PD-MSCs^PRL-1^ using lentiviral and nonviral AMAXA systems. Scale bars = 100 μm. The expression of GFP in PD-MSCs after each transfection system. Scale bars = 50 μm. **c** Western blotting of PRL-1 expression in naïve and PD-MSCs^PRL-1^. **d** RT-PCR analysis of stemness markers in naïve and PD-MSCs^PRL-1^ depending on passage. **e** FACS analysis of surface markers related to hematopoietic, nonhematopoietic, and HLA family members in PD-MSCs^PRL-1^. **f** Western blotting of Oct4 and HLA-G expression in naïve and PD-MSCs^PRL-1^. **g** H&E staining of normal and PD-MSC^PRL-1^-transplanted NOD/SCID mouse testes at 14 weeks (lenti; *n* = 2, AMAXA; *n* = 2). Scale bars = 50 μm. **h** Engraftment of PD-MSCs^PRL-1^ into mouse testes posttransplantation. Mouse tissue was used as a negative control. Human PD-MSCs were used as a positive control. Data from each group are expressed as the mean ± SD. ^*^p < 0.05 versus the Con group. Con, control; GAPDH, glyceraldehyde 3-phosphate dehydrogenase; GFP, green fluorescent protein; PRL-1, phosphatase of regenerating liver-1; TERT, telomerase reverse transcriptase

Naïve PD-MSCs at passage 8 were transfected with lentiviral and nonviral AMAXA plasmid tagged with GFP to overexpress PRL-1 in PD-MSCs (PD-MSCs^PRL-1^, PRL-1+). A positive control was used for the GFP vector. After transfection, PD-MSCs generated using lentiviral vector containing 2 μg/ml puromycin and AMAXA containing 1.5 mg/ml neomycin were stabilized in conditioned medium (Fig. 1a). In each of these systems, GFP-positive PD-MSCs^PRL-1^ had spindle-like and fibroblastoid morphologies similar to those of MSCs (Fig. 1b). Overexpression of PRL-1 was confirmed in PD-MSCs^PRL-1^ compared to naïve cells (Fig. 1c). In addition, RT-PCR results in PD-MSCs^PRL-1^ revealed the expression of stemness markers (Oct4, Nanog, and Sox2) and telomerase reverse transcriptase (TERT). In particular, human leukocyte antigen (HLA)-G, which has an immunomodulatory effect, was expressed in both cell types (Fig. 1d).

We previously confirmed the characterization of naïve PD-MSCs [27]. The immunophenotypes of both types of PD-MSCs^PRL-1^ were analyzed by flow cytometry. MSC marker clusters of differentiation (CD13, CD90, and CD105) were positive, whereas the hematopoietic and endothelial cell marker CD34 was negative in both types of PD-MSCs^PRL-1^. Moreover, HLA-DR was nearly absent. However, HLA-ABC and HLA-G were significantly positive in PD-MSCs^PRL-1^ (Fig. 1e). The protein levels of both cell types were higher than those of naïve cells (Fig. 1f). Nine-week-old male NOD/SCID mice were directly injected with PD-MSCs^PRL-1^ into the testes (*n* = 2; lentiviral, *n* = 2; AMAXA) to confirm teratoma formation in vivo. Mice were sacrificed at 14 weeks postinjection, and each testis was evaluated by H&E staining (Fig. 1g) and the human-specific Alu sequence from gDNA isolation. Testes injected with cells treated with both systems had no teratomas, and Alu expression was higher in injected testes than in noninjected testes (Fig. 1h). These findings indicated that PRL-1-overexpressing PD-MSCs were generated using lentiviral and nonviral AMAXA systems and maintained characteristics similar to those of naïve cells.

### Multidifferentiation potential of PD-MSCs^PRL-1^

**Fig. 2:**
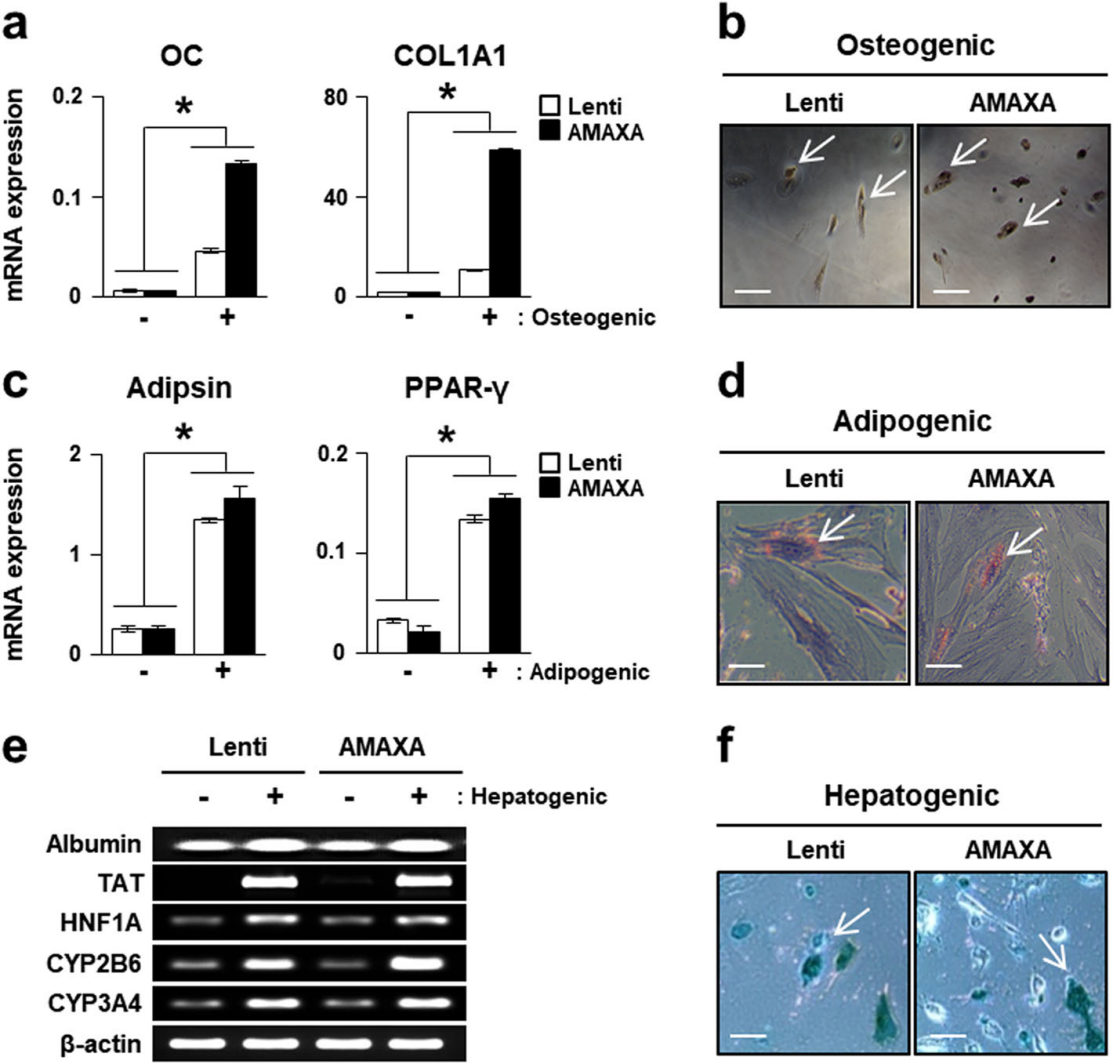
Multidifferentiation potential of PD-MSCs^PRL-1^. **a** qRT-PCR analysis of osteogenic markers (OC and COL1A1) in undifferentiated (−) and differentiated (+) PD-MSCs^PRL-1^. **b** Von Kossa staining in osteogenic differentiation of PD-MSCs^PRL-1^. Scale bars = 50 μm. **c** qRT-PCR analysis of adipogenic markers (adipsin and PPAR-γ) in undifferentiated and differentiated PD-MSCs^PRL-1^. **d** Oil Red O staining in adipogenic differentiation of PD-MSCs^PRL-1^. Scale bars = 50 μm. **e** RT-PCR analysis of hepatocyte function markers (albumin, tyrosine aminotransferase; TAT, hepatocyte nuclear factor 1 homeobox A; HNF1A, cytochrome P450 2B6; CYP2B6, and CYP3A4) in undifferentiated and differentiated PD-MSCs^PRL-1^. **f** ICG uptake in PD-MSCs^PRL-1^ after hepatogenic differentiation. Scale bars = 50 μm. Data from each group are expressed as the mean ± SD. ^*^p < 0.05 versus undifferentiated group. COL1A1, collagen type 1 alpha 1; CYP2B6, cytochrome P450 2B6; HNF1A, hepatocyte nuclear factor 1 homeobox A; OC, osteocalcin; PPAR-γ, peroxisome proliferator-activated receptor gamma; TAT, tyrosine aminotransferase

PD-MSCs^PRL-1^ that differentiated into mesodermal lineages (osteogenic and adipogenic) were identified using von Kossa and Oil Red O staining (Fig. 2b, d). qRT-PCR revealed that the expression of osteogenic-specific markers (osteocalcin; OC and collagen type 1 alpha 1; COL1A1) was increased in differentiated compared with undifferentiated PD-MSCs^PRL-1^ (Fig. 2a). In addition, the expression of adipsin and peroxisome proliferator-activated receptor gamma (PPAR-γ), which are specific markers for adipocytes, was higher in differentiated cells than in undifferentiated cells (Fig. 2c). Furthermore, both types of PD-MSCs^PRL-1^ differentiated into endodermal lineage hepatocytes. The mRNA expression of hepatogenic markers, including albumin, tyrosine aminotransferase (TAT), hepatocyte nuclear factor 1 homeobox A (HNF1A), cytochrome P450 2B6 (CYP2B6), and CYP3A4 was higher than differentiated PD-MSCs^PRL-1^ than undifferentiated cells using both gene delivery systems (Fig. 2e). ICG uptake was evaluated in lentiviral and nonviral AMAXA PD-MSCs^PRL-1^ (Fig. 2f) to determine whether these cells exhibited hepatocyte function. These findings indicate that both types of PD-MSCs^PRL-1^ retain the ability to differentiate into mesoderm and endodermal lineage cells.

### Effect of PRL-1-dependent migration ability through the Rho family

**Fig. 3:**
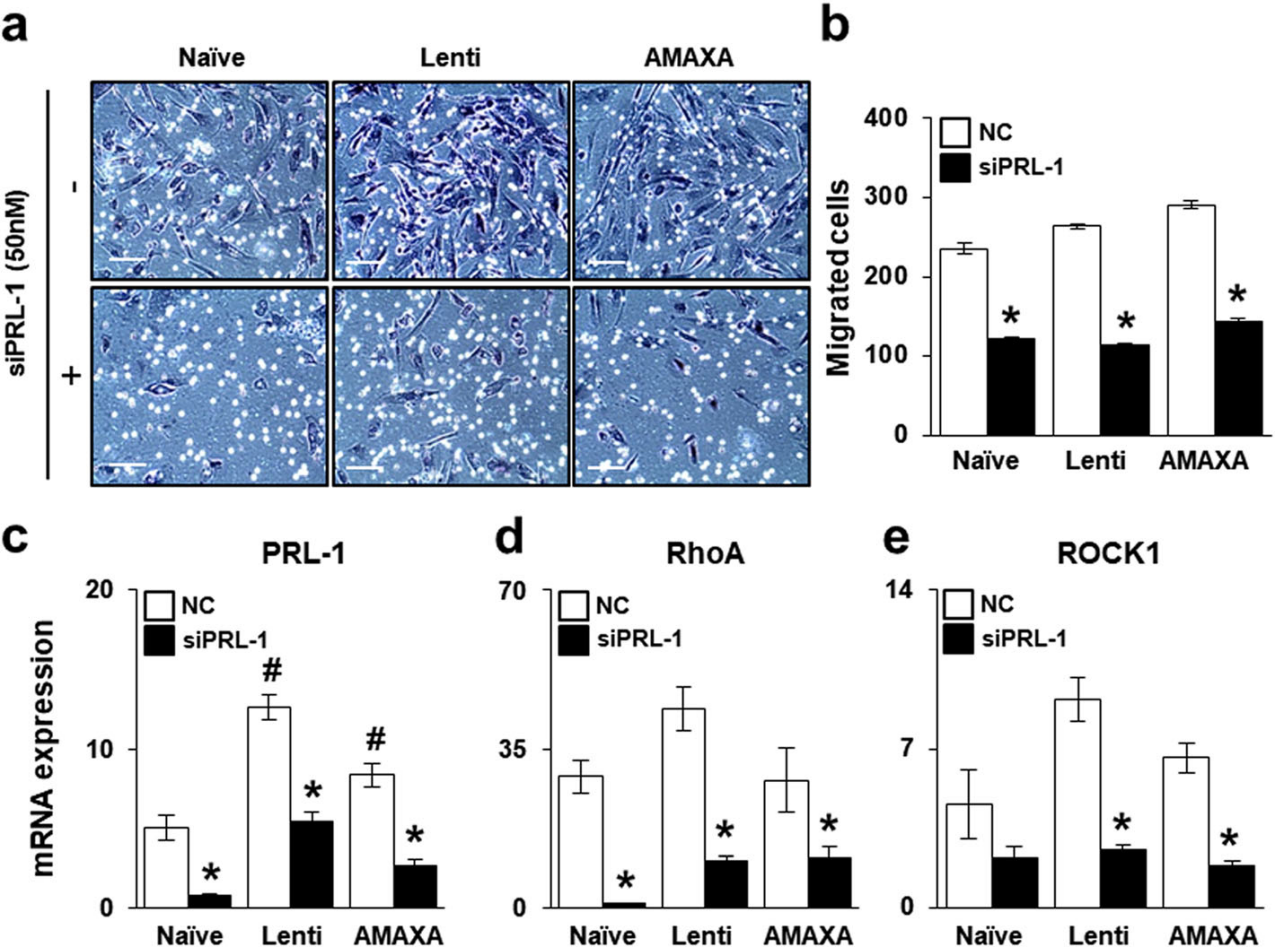
Effect of PRL-1-dependent migration ability through the Rho family. **a**, **b** Representative images and quantification of migrated naïve and PD-MSCs^PRL-1^ in a Transwell insert system treated or untreated with siRNA-PRL-1 (siPRL-1; 50 nM) after 24 h. Scale bars = 100 μm. qRT-PCR analysis of **c** PRL-1, **d** RhoA, and **e** ROCK1 expression in migrated naïve and PD-MSCs^PRL-1^ from the upper chamber to the lower chamber after treatment with or without siPRL-1. Data from each group are expressed as the mean ± SD. ^*^p < 0.05 versus NC; ^#^*p* < 0.05 versus naïve. NC, negative control; PRL-1, phosphatase of regenerating liver-1

To analyze the effect of PRL-1 on the migration ability of PD-MSCs^PRL-1^, we performed a migration assay with PD-MSCs^PRL-1^ using a Transwell insert system. The number of both types of migrated PD-MSCs^PRL-1^ was higher than that of naïve cells (Fig. 3a). In addition, the number of migrated naïve and PD-MSCs^PRL-1^ was significantly decreased when cocultured with siRNA-PRL-1 treatment (Fig. 3b). PRL-1 expression as well as RhoA and ROCK1 expression was increased in PD-MSCs^PRL-1^ compared with naïve cells. Alternatively, downregulated PRL-1 decreased migration ability (Fig. 3c–e). Our data suggest that PRL-1 may regulate migration ability in a dependent manner.

### Improved mitochondrial respirational metabolic states of PD-MSCs^PRL-1^ regulate mitochondrial biogenesis

**Fig. 4:**
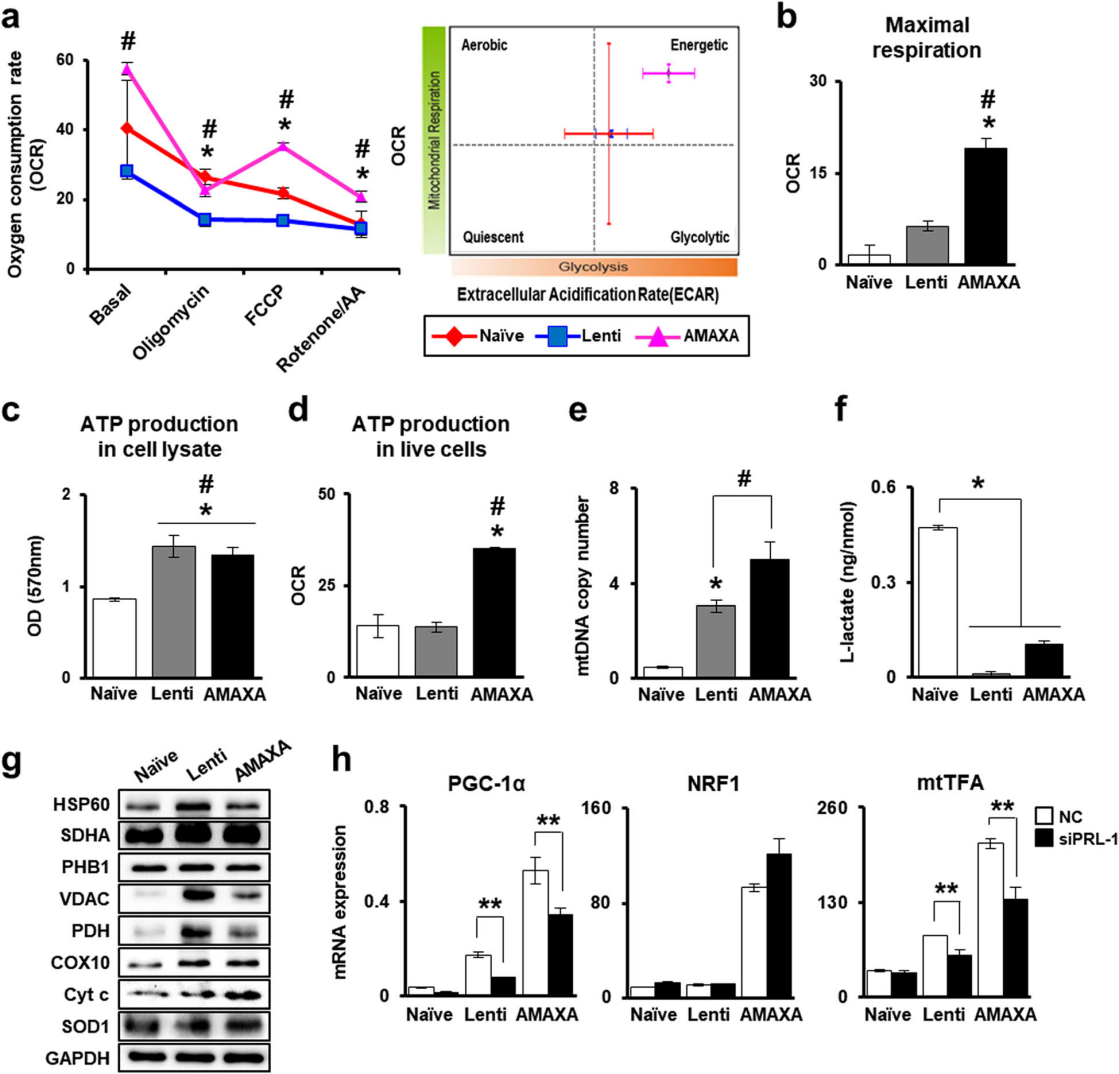
Improved mitochondrial respirational metabolic states of PD-MSCs^PRL-1^ regulate mitochondrial dynamics. **a** OCR and ECAR of PD-MSCs^PRL-1^ using lentiviral and nonviral AMAXA systems after sequential treatment with 1 μM oligomycin, 0.5 μM FCCP, and 0.5 μM rotenone/AA by the Seahorse XF24 Analyzer. Quantification of **b** maximal respiration and **d** ATP production under live conditions of naïve and PD-MSCs^PRL-1^. **c** ATP production assay in naïve and PD-MSC^PRL-1^ lysate (10 μg/μl). **e** mtDNA copy number of PD-MSCs^PRL-1^ using lentiviral and nonviral AMAXA systems by the TaqMan assay. Human nuclear DNA was used as an internal control. **f** L-lactate production assay of PD-MSCs^PRL-1^ using cell lysate (< 25 mg/ml). **g** Western blotting of mitochondrial metabolism-specific genes in naïve and PD-MSCs^PRL-1^. **h** qRT-PCR analysis of mitochondrial biogenesis-specific genes (PGC-1α, NRF1, and mtTFA) in PD-MSC^PRL-1^-treated or -untreated siRNA-PRL-1 (siPRL-1; 50 nM) after 24 h. Data from each group are shown as the mean ± SD. ^*^p < 0.05 versus naïve; ^#^*p* < 0.05 versus lenti; ^**^p < 0.05 versus NC. AA, antimycin A; COX10, cytochrome c oxidase 10; Cyt c, cytochrome c; ECAR, extracellular acidification rate; FCCP, carbonyl cyanide-p-trifluoromethoxyphenylhydrazone; GAPDH, glyceraldehyde 3-phosphate dehydrogenase; HSP60, heat shock protein 60; mtTFA, mitochondrial transcription factor A; NC, negative control; NRF1, nuclear respiratory factor 1; OCR, oxygen consumption rate; PDH, pyruvate dehydrogenase; PGC-1α, peroxisome proliferator-activated receptor gamma coactivator-1 alpha; PHB1, prohibitin 1; SDHA, succinate dehydrogenase; SOD1, superoxide dismutase 1; VDAC, voltage-dependent anion channel

To analyze mitochondrial respiration in PD-MSCs^PRL-1^ according to both gene delivery systems, we used the XF Cell Mito Stress test to evaluate the impact of mitochondrial complex inhibitors (oligomycin, FCCP, and rotenone/AA) on the electron transport chain (ETC). In particular, PD-MSCs^PRL-1^ generated using the nonviral system had a more energetic metabolic state than did the cells in the naïve and lentiviral groups (Fig. 4a). The maximal respiration capacity, which may account for the capability of cells to respond to an energy demand, was increased in PD-MSCs^PRL-1^ compared to naïve cells. In particular, nonviral system-based PD-MSCs^PRL-1^ showed significant improvement in this capacity (Fig. 4b). In whole-cell lysates, ATP, the mitochondrial final product, was also relatively increased in PD-MSCs^PRL-1^ generated using both systems compared with that in naïve cells (Fig. 4c).

Interestingly, although naïve and PD-MSCs^PRL-1^ using lentiviral groups showed no differences, intracellular ATP production in live cells in the nonviral AMAXA system group markedly improved (Fig. 4d). mtDNA copy number was confirmed to isolate gDNA from naïve and PD-MSCs^PRL-1^ and investigate self-renewal ability through mitochondrial function. mtDNA copy number was higher in PD-MSCs^PRL-1^ than in naïve cells (Fig. 4e). In particular, PD-MSCs^PRL-1^ in the AMAXA system group prominently had a higher mtDNA copy number. The production of L-lactate, the end product of glycolysis, was assayed. L-lactate levels decreased in PD-MSCs^PRL-1^ generated using both systems compared with those in naïve cells (Fig. 4f). In glycolysis, pyruvate that forms under aerobic conditions undergoes conversion to acetyl Co-A by pyruvate dehydrogenase (PDH). Although heat shock protein 60, which mediates protein folding after import into the mitochondria, and prohibitin 1 (PHB1), which exhibits membrane-bound ring complex expression, showed no differences, the protein expression of PDH was higher in enhanced PD-MSCs^PRL-1^ generated using both systems than in naïve systems. In mitochondria, to produce ATP, succinate dehydrogenase (SDHA), a key component in Complex II of the ETC, increased in PD-MSCs^PRL-1^ generated using lentiviral and nonviral systems. The expression of cytochrome c (cyt c) related to Complex 4 of the ETC and cyt c oxidase (COX10) also increased in PD-MSCs^PRL-1^. In addition, through voltage-dependent anion channels (VDAC) located in the mitochondrial outer membrane, superoxide free radicals are rapidly converted to H_2_O_2_ by superoxide dismutase 1 (SOD1) in the aerobic system [28].

Although VDAC expression in PD-MSCs^PRL-1^ was markedly higher than that in naïve cells, SOD1 showed no differences (Fig. 4g and Supplementary Fig. 1). In mammals, improvement in aerobic metabolic capacity occurs through transcriptionally regulated mitochondrial biogenesis [29]. The major factors in biogenesis (PGC-1α, nuclear respiratory factor 1; NRF1 and mtTFA) were detected by mRNA levels. Although NRF1 expression showed no differences following downregulation of PRL-1, PGC-1α and mtTFA expression significantly decreased after PRL-1 knockdown (Fig. 4h). These results suggest that enhanced PRL-1 in PD-MSCs using both gene delivery systems may affect mitochondrial biogenesis, particularly PRL-1 induced by the nonviral AMAXA system, promoting cellular aerobic metabolism.

### In vivo nonviral PD-MSCs^PRL-1^ alleviate liver fibrosis in a rat model of BDL

**Fig. 5:**
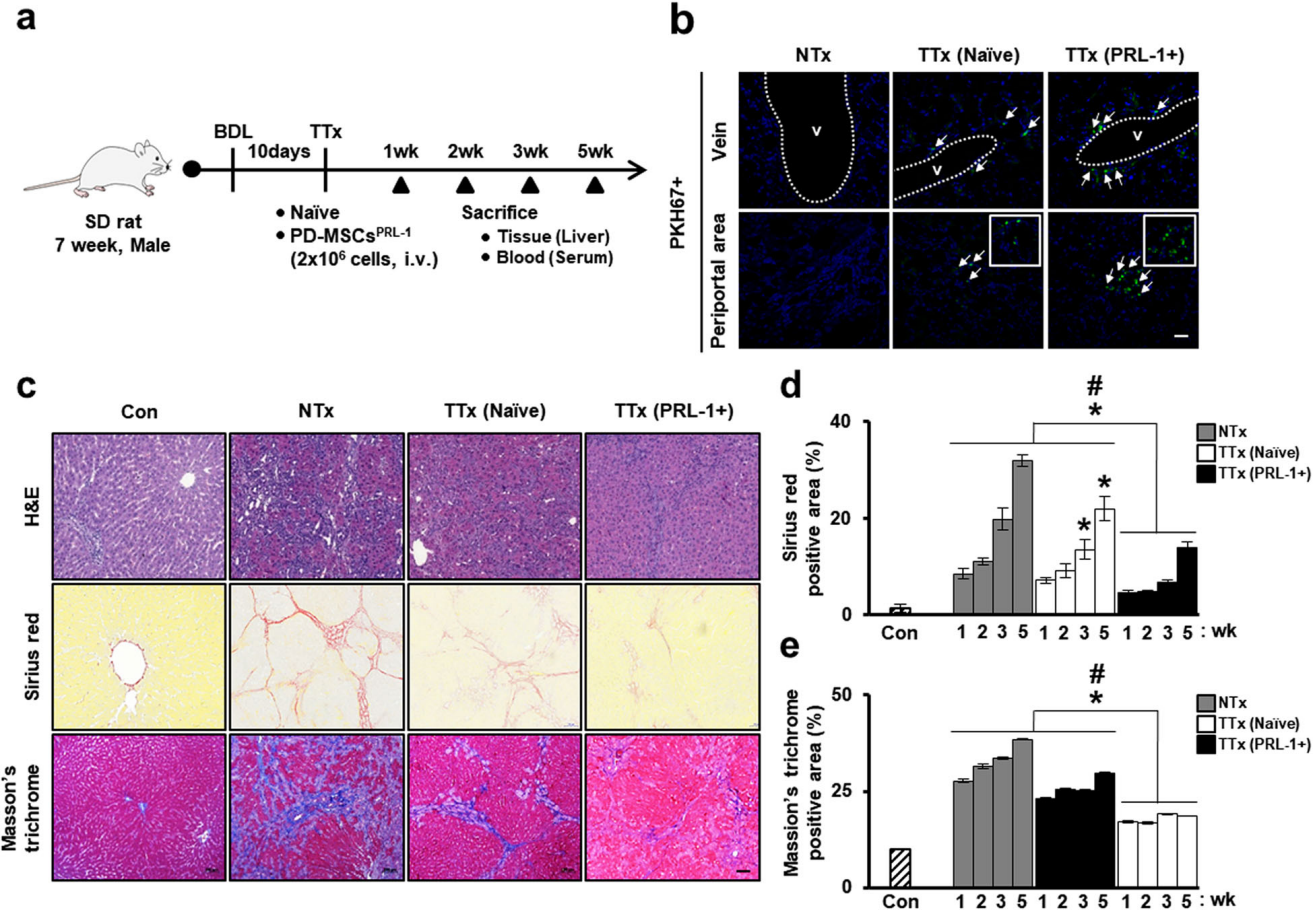
In vivo nonviral PD-MSCs^PRL-1^ alleviate liver fibrosis in a rat BDL model. **a** A schematic diagram describing the rat BDL model (NTx; *n* = 20, TTx naïve; *n* = 20, TTx PRL-1+; *n* = 20) as well as sham controls (Con; *n* = 5). **b** Engraftment of PKH67 (green)-labeled transplanted cells as injured rat liver by fluorescence microscopy at 1 week (arrow: PKH67+ signals) (green: PKH67; blue: DAPI). Scale bars = 100 μm. **c** Representative images of histopathological analysis in rat liver sections stained with H&E, Sirius red, and Masson’s trichrome after naïve and PD-MSC^PRL-1^ transplantation at 3 weeks. Scale bars = 100 μm. Quantification of **d** Sirius red- and **e** Masson’s trichrome-positive areas at 1, 2, 3, and 5 weeks in stained liver slides (*n* = 5). Data from each group are presented as the mean ± SD. ^*^p < 0.05 versus NTx group; ^#^*p* < 0.05 versus TTx naïve group. BDL, bile duct ligation; Con, control group; H&E, hematoxylin and eosin; NTx, nontransplantation group; PRL-1, phosphatase of regenerating liver-1; TTx, transplantation group

BDL is the most common model of cholestasis injury in rodents and results in inflammation, hepatocyte apoptosis and fibrosis [30]. To analyze the hepatic regeneration of PD-MSCs^PRL-1^ generated using a nonviral AMAXA system in the BDL model, we established an early cirrhotic model. Ten days after BDL, we distributed the animals into the following groups: normal control group (Con), nontransplantation group (NTx), naïve PD-MSC transplantation group (TTx naïve), and PD-MSC^PRL-1^ transplantation group (TTx PRL-1+) (Fig. 5a). To evaluate the intrahepatic distribution of transplanted cells, PKH67+ cells were identified among the vein and periportal fibrotic area in the liver sections of TTx naïve and PRL-1+ groups at 1 week (Fig. 5b). In comparison to the livers in the normal control rats, those in the BDL-induced NTx group exhibited diffuse bile ductular proliferation with loss of enlarged hepatocytes. Histopathological analysis determined that the collagen fibrotic area was distinctly increased. Compared with the NTx rats, the transplanted rats showed decreased Sirius red and Masson’s trichrome-stained collagen fiber areas, and their hepatocytes were partly replaced by ductules at 3 weeks (Fig. 5c). In particular, compared with the TTx naïve group rats, the transplanted TTx PRL-1+ rats exhibited significantly decreased quantification of the fibrotic area based on Sirius red and Masson’s trichrome staining (Fig. 5d, e). These findings indicate that administration of PD-MSCs^PRL-1^ induced by a nonviral AMAXA gene delivery system decreased liver fibrosis in a rat BDL model.

### PD-MSCs^PRL-1^ from a nonviral system enhance hepatic function by regulating mitochondrial metabolism in a rat BDL model

**Fig. 6:**
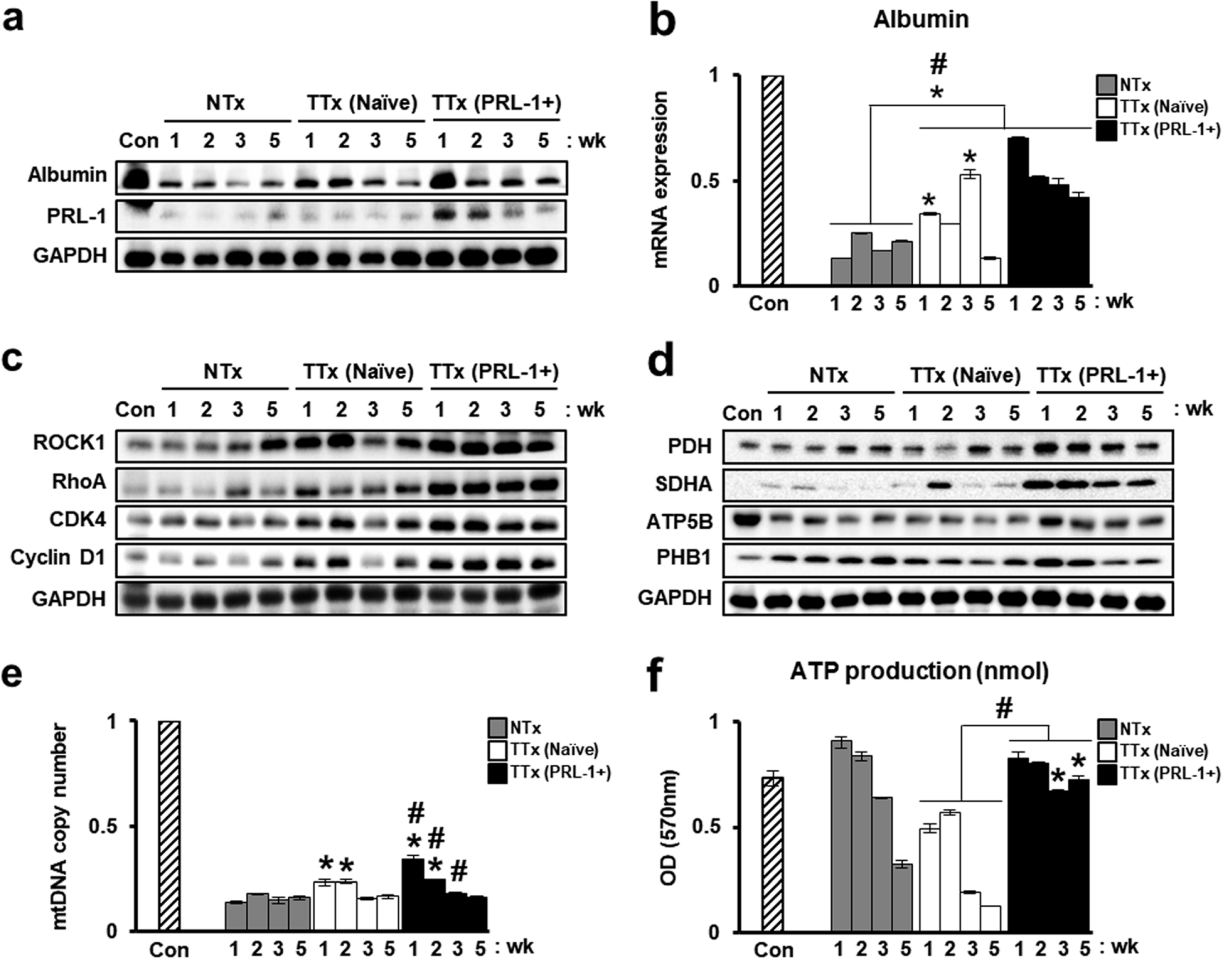
PD-MSCs^PRL-1^ using a nonviral system enhances hepatic function by regulating mitochondrial metabolism in a rat BDL model. **a** Western blotting of albumin and human PRL-1 gene expression by PD-MSC^PRL-1^ transplantation in pooled BDL-injured rat liver tissue (*n* = 5). **b** mRNA expression of albumin in a rat model with pooled BDL liver tissue (*n* = 5). Protein expression of the **c** Rho family (ROCK1 and RhoA), cell cycle (CDK4 and Cyclin D1), and mitochondrial metabolism (PDH, SDHA, ATP5B, and PHB1) by PD-MSC^PRL-1^ transplantation in pooled BDL-injured rat liver tissue (*n* = 5). **e** mtDNA copy number in BDL-injured rat liver tissue by the TaqMan assay. Rat nuclear DNA was used as an internal control. **f** ATP production assay in rat BDL liver lysate (10 μg/μl). Data from each group are presented as the mean ± SD. ^*^p < 0.05 versus NTx group; ^#^*p* < 0.05 versus TTx naïve group. CDK4; cyclin-dependent kinase 4, Con, control group; GAPDH, glyceraldehyde 3-phosphate dehydrogenase; NTx, nontransplantation group; PDH, pyruvate dehydrogenase; PHB1, prohibitin 1; PRL-1, phosphatase of regenerating liver-1; SDHA, succinate dehydrogenase; TTx, transplantation group

mRNA and protein expression of albumin were increased in the transplantation groups compared with the NTx group, and these findings confirmed hepatic function by PD-MSC^PRL-1^ transplantation in BDL-injured rat livers. Albumin levels as well as human PRL-1 expression were remarkably higher in the TTx PRL-1+ group than in the TTx naïve group at 1 week (Fig. 6a, b). In addition, increased serum levels of ALT, AST, and TBIL and decreased albumin levels were confirmed in the NTx group compared to the Con group. Although TBIL levels in TTx PRL-1+ livers showed no differences compared with TTx naïve livers, the serum levels of ALT, AST, and albumin were markedly improved (Table 4). The protein expression of RhoA and ROCK1 was increased in the TTx groups compared with that in the NTx group. Interestingly, the TTx PRL-1 group showed significantly higher expression of CDK4 and Cyclin D1 than did the TTx naïve group (Fig. 6c). To demonstrate alterations in hepatic metabolism induced by PD-MSCs^PRL-1^ in BDL, the protein levels of mitochondrial metabolism-specific markers (PDH, SDHA, ATP5B, and PHB1) were significantly increased in the TTx PRL-1+ group compared with the NTx and TTx naïve groups (Fig. 6d). Moreover, mtDNA copy number and ATP production in the naïve transplantation group were higher than those in the NTx group. In particular, compared with the TTx naïve group, the TTx PRL-1+ group showed remarkably enhanced mtDNA copy number and ATP production (Fig. 6e, f). These findings indicate that PD-MSCs^PRL-1^ induced by a nonviral AMAXA gene delivery system may promote hepatic function by regulating mitochondrial metabolism in the liver.

**Table 4:** Improved liver functions according to MSC transplantation in rat serum levels of BDL model

	ALT (U/L)	AST (U/L)	TBIL (μmol/L)	Albumin (g/L)
Normal control (Con)	39.4 ± 3.6	97.1 ± 7.3	1.1 ± 0.3	34.4 ± 2.3
NTx	152.8 ± 7.2^†^	797.4 ± 32.0^†^	121.1 ± 4.8^†^	21.5 ± 0.5^†^
TTx (naïve)	125.6 ± 6.9^†, *^	690.9 ± 34.7^†, *^	96.5 ± 5.3^†, *^	24.2 ± 0.5^†, *^
TTx (PRL-1+)	104.0 ± 9.1^†, *, #^	523.3 ± 34.0^†, *, #^	101.4 ± 1.7^†, *^	27.0 ± 0.3^†, *, #^

Each group was *n* = 18. Data are presented as mean ± SD. ^†^*p* < 0.05 versus Con group; ^*^p < 0.05 versus NTx group; ^#^*p* < 0.05 versus TTx naïve group. *ALT*; alanine aminotransferase, *AST*; aspartate aminotransferase, *Con*; control group, *TBIL*; total bilirubin

### PD-MSC^PRL-1^ coculture activates the mitochondrial dynamics of primary hepatocytes

**Fig. 7:**
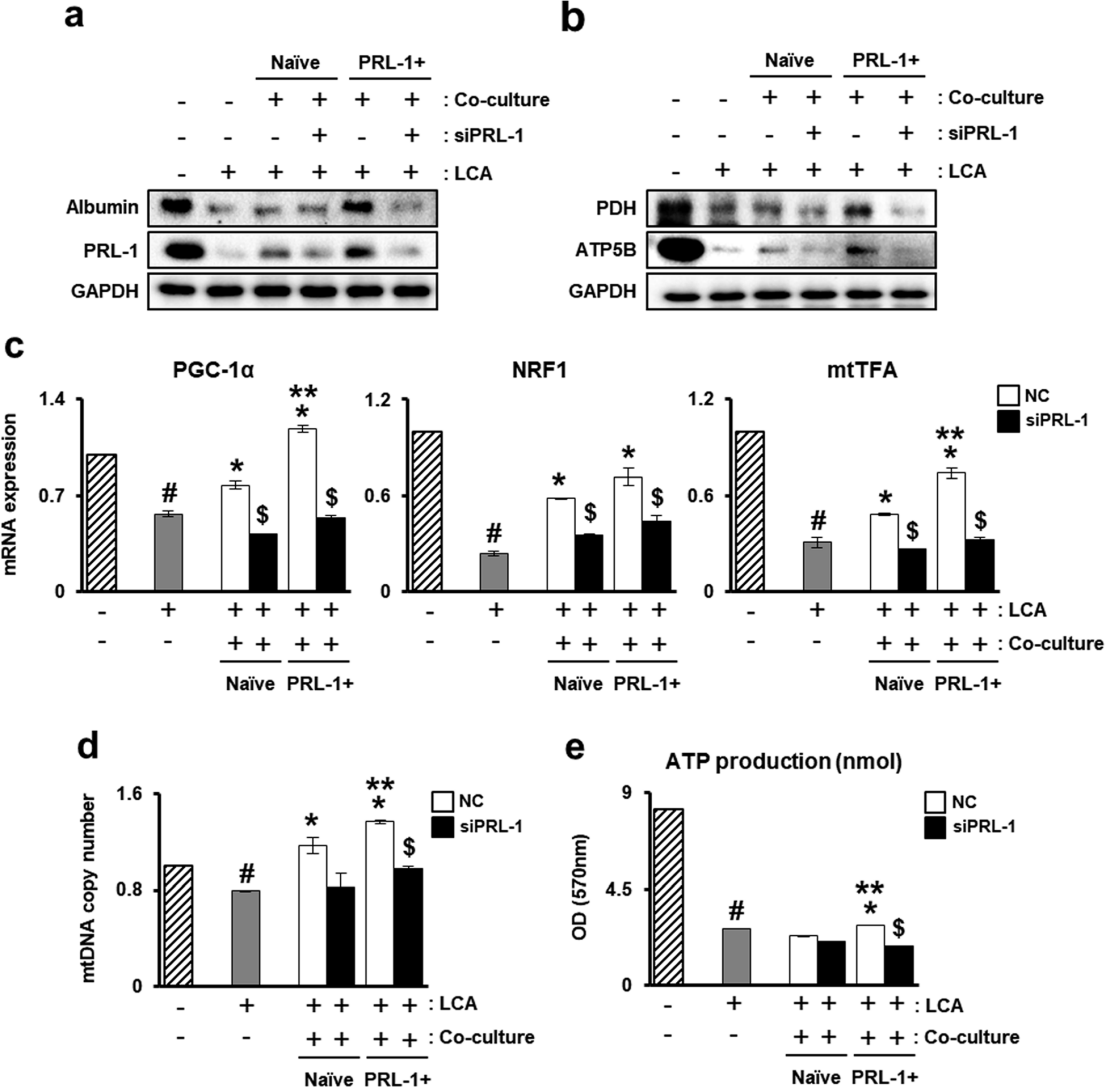
PD-MSC^PRL-1^ coculture activates mitochondrial dynamics of primary hepatocytes. Protein expression of **a** albumin and PRL-1 and **b** mitochondrial metabolism (PDH and ATP5B) by naïve or PD-MSC^PRL-1^ coculture with or without siRNA-PRL-1 (siPRL-1; 50 nM) for 24 h in primary hepatocytes treated with lithocholic acid (LCA; 100 μM) for 12 h by western blotting. **c** mRNA expression of mitochondrial dynamics (PGC-1α, NRF1, and mtTFA) by qRT-PCR analysis. **d** mtDNA copy number in primary hepatocytes by the TaqMan assay. **e** ATP production analysis in primary hepatocyte lysate (10 μg/μl). Data from each group are presented as the mean ± SD. ^#^*p* < 0.05 versus control (−); ^*^p < 0.05 versus coculture groups; ^**^p < 0.05 versus naïve; ^$^*p* < 0.05 versus NC. GAPDH, glyceraldehyde 3-phosphate dehydrogenase; mtTFA, mitochondrial transcription factor A; NC, negative control; NRF1, nuclear respiratory factor 1; PDH, pyruvate dehydrogenase; PGC-1α, peroxisome proliferator-activated receptor gamma coactivator-1 alpha; PRL-1, phosphatase of regenerating liver-1

To demonstrate hepatic mitochondrial function, primary hepatocytes were isolated from rats and cocultured with nonviral PD-MSCs^PRL-1^ in accordance with PRL-1 knockdown. In vitro modeling was induced by LCA treatment for the accumulation of bile acid. In advance, the protein expression of albumin as well as that of PRL-1 in hepatocytes was decreased according to LCA treatment. However, compared to naïve cells, PD-MSC^PRL-1^ coculture recovered the expression of albumin and PRL-1 (Fig. 7a). To verify the mitochondrial function of hepatocytes, although the protein expression of mitochondrial metabolism (PDH and ATP5B) decreased with LCA treatment, naïve PD-MSC coculture was slightly increased. Interestingly, PDH and ATP5B levels in PD-MSC^PRL-1^ coculture were higher than those in naïve cells (Fig. 7b). In addition, the mRNA levels of PGC-1α, NRF1, and mtTFA, which are key factors in mitochondrial dynamics, were decreased in the LCA treatment group. However, PD-MSC^PRL-1^ coculture significantly improved gene expression (Fig. 7c). mRNA and protein expression was PRL-1-dependent. While mtDNA copy number and ATP production in primary hepatocytes receiving LCA treatment were reduced, compared to naïve cells, PD-MSC^PRL-1^ coculture led to a significant increase (Fig. 7d, e). These data suggest that PD-MSCs^PRL-1^ may stimulate improved mitochondrial metabolism in primary hepatocytes.

## Discussion

As an alternative to liver transplantation, MSC-based therapy for regenerative medicine has been attempted for the treatment of various liver diseases, including hepatic failure and liver cirrhosis [31, 32]. According to our previous reports, PD-MSCs have antifibrotic effects by increased MMP activities as well as hepatic regeneration in a CCl_4_-injury rat model via an increased autophagic mechanism [27]. Recently, researchers have attempted clinical trials in patients with alcoholic hepatic cirrhosis using autologous BM-MSCs. In phase I and II clinical trials, Jang and colleagues reported that BM-MSCs were primarily safe and that autologous BM-MSCs had antifibrotic effects in patients with alcoholic hepatic cirrhosis [33]. Nevertheless, clinical applications of BM-MSCs have been hampered by several weaknesses, including a painful isolation procedure for patients, a lack of fresh cells, a high price, and a lack of understanding of the therapeutic mechanism [34]. The production of functionally enhanced MSCs with therapeutic efficacy-enhancing factors and verification of therapeutic efficacy are emerging to overcome these limitations of MSC-based cell therapy.

Recently, PRL-1 has been shown to play an important role in cell cycle progression during hepatic regeneration [22]. However, the effect of PRL-1 on PD-MSC properties remains poorly understood. In our study, we demonstrated that PRL-1-overexpressing PD-MSCs (PD-MSCs^PRL-1^, PRL-1+) were successfully generated using lentiviral and nonviral AMAXA gene delivery systems, and we analyzed their characteristics, which were similar to those of naïve cells (Figs. 1 and 2). Interestingly, the mitochondrial respirational metabolic state and cellular aerobic metabolism significantly increased in PD-MSCs^PRL-1^ in both gene delivery systems. In particular, mtDNA synthesis and mitochondrial biogenesis were remarkably enhanced by PRL-1 in the nonviral AMAXA system, and ATP production in mitochondria in live cellular conditions also increased, maintaining an energetic state (Fig. 4). Although differences in cellular mitochondrial mechanisms according to gene delivery systems have remained unclear, viral systems carry a possible risk of cancer and immunologic side effects [35].

In general, MSCs genetically engineered by viral gene delivery systems have important safety issues, such as the genomic instability of the cells. For this reason, many researchers have attempted to produce stem cells with enhanced function based on nonviral gene delivery systems, such as AMAXA systems, and have also applied them to clinical trials using enhanced functional stem cells. In the present study, PD-MSCs^PRL-1^ were generated using nonviral AMAXA systems, and no teratoma formation occurred. Our studies revealed that compared with naïve PD-MSC transplantation, nonviral PD-MSC^PRL-1^ transplantation remarkably enhanced engraftment in the injured target organs through the activated Rho family and albumin synthesis (Fig. 6). These data are similar to previous studies demonstrating that PRL-1 binding to p115 Rho GAP promotes ERK1/2 and RhoA activation [19].

**Fig. 8:**
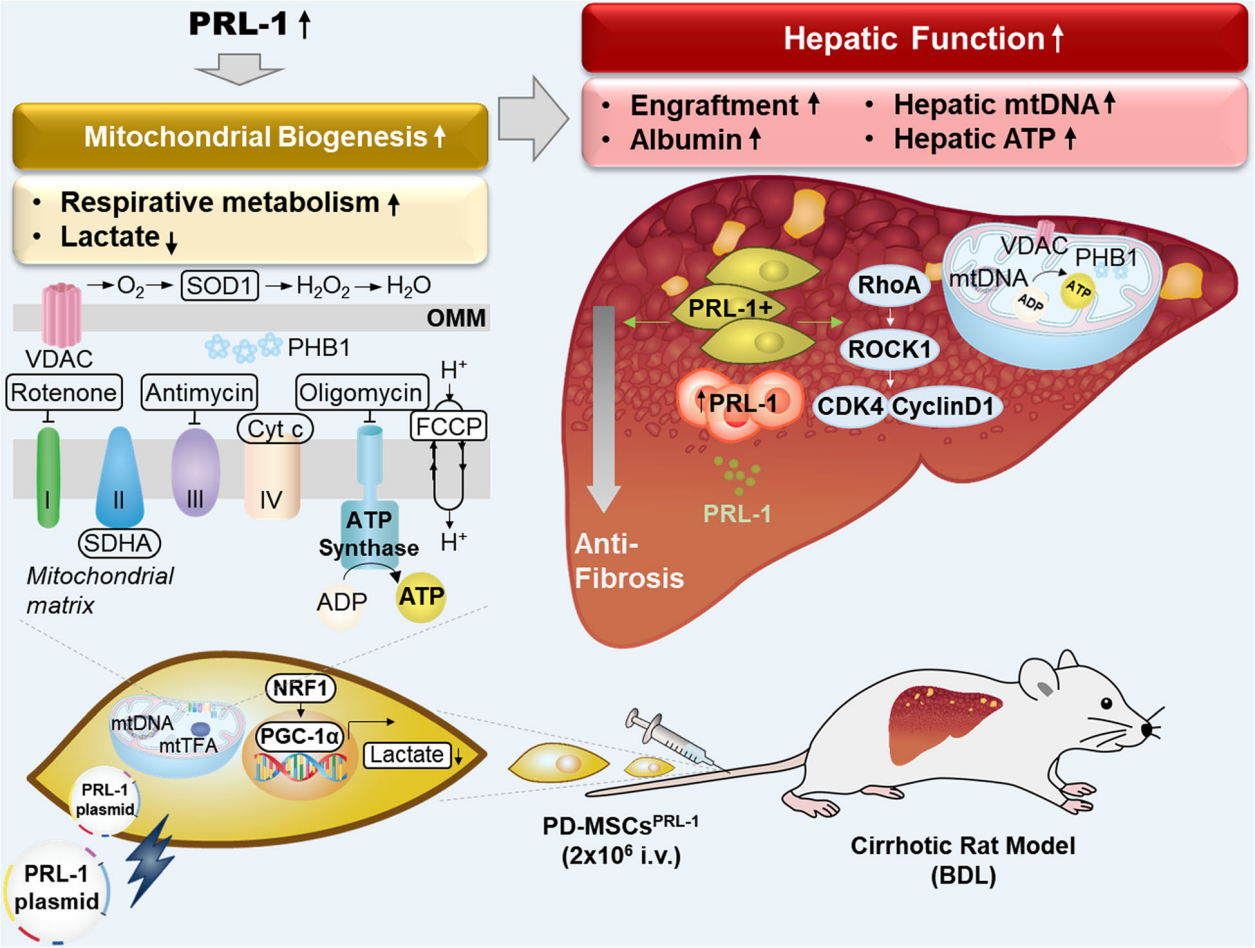
Summarized diagram proposes that PD-MSCs^PRL-1^ using the nonviral AMAXA system promote mitochondrial respirational states and accelerate liver regeneration by improving hepatic mitochondrial functions. BDL, bile duct ligation; CDK4, cyclin-dependent kinase 4; Cyt c, cytochrome c; FCCP, carbonyl cyanide-p-trifluoromethoxyphenylhydrazone; I.V., intravenous; mtDNA, mitochondrial DNA; mtTFA, mitochondrial transcription factor A; NRF1, nuclear respiratory factor 1; PD-MSCs, placenta-derived mesenchymal stem cells; PGC1α, peroxisome proliferator-activated receptor gamma coactivator-1 alpha; PHB1, prohibitin 1; PRL-1, phosphatase of regenerating liver-1; SDHA, succinate dehydrogenase; VDAC, voltage-dependent anion channel

Mitochondria-mediated function is associated with liver regeneration [36]. In chronic cholestasis, mitochondrial dysfunction, associated with decreased activities of respiratory chain complexes, mitochondrial membrane potential, and ATP production, was characterized [37]. The lack of stimulation of mitochondrial biogenesis in a rat BDL model leads to severe depletions and deletions of mtDNA copy number [38]. Additionally, hepatic injury caused by cholestasis affects oxidative stress in mitochondria in the liver. PGC-1α and mtTFA are impaired in the BDL model [39]. By transplantation of MSCs, hepatic mitochondrial dysfunctions are reversed by mediating the nuclear factor erythroid-derived 2-related factor 2 (Nrf2)/HO1 pathway [40]. The effects of MSCs are due to the more rapid recovery of the number of mitochondria and the function of hepatic mitochondria [41]. These previous reports support our conclusion that mtDNA and ATP synthesis are increased in liver tissues during liver regeneration following transplantation of functionally enhanced PD-MSCs. PD-MSCs^PRL-1^ generated using nonviral AMAXA system transplantation promoted mtDNA synthesis. Additionally, to promote hepatic ATP production, PDH and SDHA are more activated in hepatic mitochondrial ETCs in PD-MSCs^PRL-1^ than in naïve cells. Finally, hepatic ATP production was enhanced by PD-MSCs^PRL-1^ generated using nonviral AMAXA system transplantation in a rat BDL model as well as primary hepatocytes (Figs. 7 and 8). Transplantation of PD-MSCs with PRL-1 using a nonviral AMAXA system in a BDL rat hepatic disease model promotes hepatic functions by upregulating mitochondrial metabolism.

## Conclusion

In conclusion, PD-MSCs^PRL-1^ generated using the nonviral AMAXA system impacted mitochondrial respirational metabolic states more than those generated using the lentiviral-based gene delivery system. In particular, the therapeutic effects of PD-MSCs^PRL-1^ using the nonviral AMAXA system accelerate liver function by promoting mitochondrial metabolism (Fig. 8). These findings provide novel insights into next-generation MSC-based cell therapy, including safe gene modification, and useful data for therapeutic strategies using PD-MSCs for regenerative medicine in liver disease.

## Supplementary Information

**Additional file 1: Supplementary Fig. 1** Quantification of mitochondrial metabolism-specific genes in naïve and PD-MSCs^PRL-1^ using both gene delivery systems. Data from each group are shown as the mean ± SD. ^*^p < 0.05 versus naïve; ^#^*p* < 0.05 versus lenti. *COX10*; cytochrome c oxidase 10, *Cyt c*; cytochrome c, *GAPDH*; glyceraldehyde 3-phosphate dehydrogenase, *HSP60*; heat shock protein 60, *PDH*; pyruvate dehydrogenase, *PHB1*; prohibitin 1, *SDHA*; succinate dehydrogenase, *SOD1*; superoxide dismutase 1, *VDAC*; voltage-dependent anion channel.

## Data Availability

All data analyzed for this study are included in this article.

## Abbreviations

AA: Antimycin A
ALT: Alanine aminotransferase
α-MEM: Alpha-modified minimal essential medium
AST: Aspartate aminotransferase
BDL: Bile duct ligation
bFGF: Basic fibroblast growth factor
BM-MSCs: Bone marrow-derived mesenchymal stem cell
CCl_4_: Carbon tetrachloride
CD: Cluster of differentiation
COL1A1: Collagen type 1 alpha 1
COX10: Cytochrome c oxidase
Cyt c: Cytochrome c
CYP2B6: Cytochrome P450 2B6
DAPI: 4′,6-Diamidino-2-phenylindole
DMEM: Dulbecco’s modified Eagle medium
ECAR: Extracellular acidification rate
EGF: Epidermal growth factor
ETC: Electron transport chain
FBS: Fetal bovine serum
FCCP: Carbonyl cyanide-*p*-trifluoromethoxyphenylhydrazone
GAPDH: Glyceraldehyde 3-phosphate dehydrogenase
gDNA: Genomic DNA
GFP: Green fluorescent protein
hFGF-4: Human fibroblast growth factor-4
H&E: Hematoxylin and eosin
HLA: Human leukocyte antigen
HNF1A: Hepatocyte nuclear factor 1 homeobox A
HO: Heme oxygenase
ICG: Indocyanine green
IL-6: Interleukin-6
IRB: Institutional Review Board
MMP: Matrix metalloproteinase
mtDNA: Mitochondrial DNA
mtTFA: Mitochondrial transcription factor A
NBF: Neutral buffered formalin
NOD/SCID: Nonobese diabetic/severe combined immunodeficiency
NRF1: Nuclear respiratory factor 1
Nrf2: Nuclear factor erythroid-derived 2-related factor 2
OC: Osteocalcin
OCR: Oxygen consumption rate
PDH: Pyruvate dehydrogenase
PD-MSCs: Placenta-derived mesenchymal stem cells
PGC-1α: Peroxisome proliferator-activated receptor gamma coactivator-1 alpha
PHB1: Prohibitin 1
PPAR-γ: Peroxisome proliferator-activated receptor gamma
PRL-1: Phosphatase of regenerating liver-1
P/S: Penicillin/streptomycin
PTP4A1: Protein tyrosine phosphatase type 4 A member 1
SD: Sprague-Dawley
SDHA: Succinate dehydrogenase
SOD1: Superoxide dismutase 1
STAT3: Signal transducer and activator of transcription 3
TAT: Tyrosine aminotransferase
TBIL: Total bilirubin
TERT: Telomerase reverse transcriptase
VDAC: Voltage-dependent anion channel
