# Supplementary figures and images for: Enhanced PRL-1 expression in placenta-derived mesenchymal stem cells accelerates hepatic function via mitochondrial dynamics in a cirrhotic rat model
Source: Stem Cell Res Ther. 2020 Nov 27;11:512. doi: 10.1186/s13287-020-02029-3 (PMC7694436; doi:10.1186/s13287-020-02029-3)

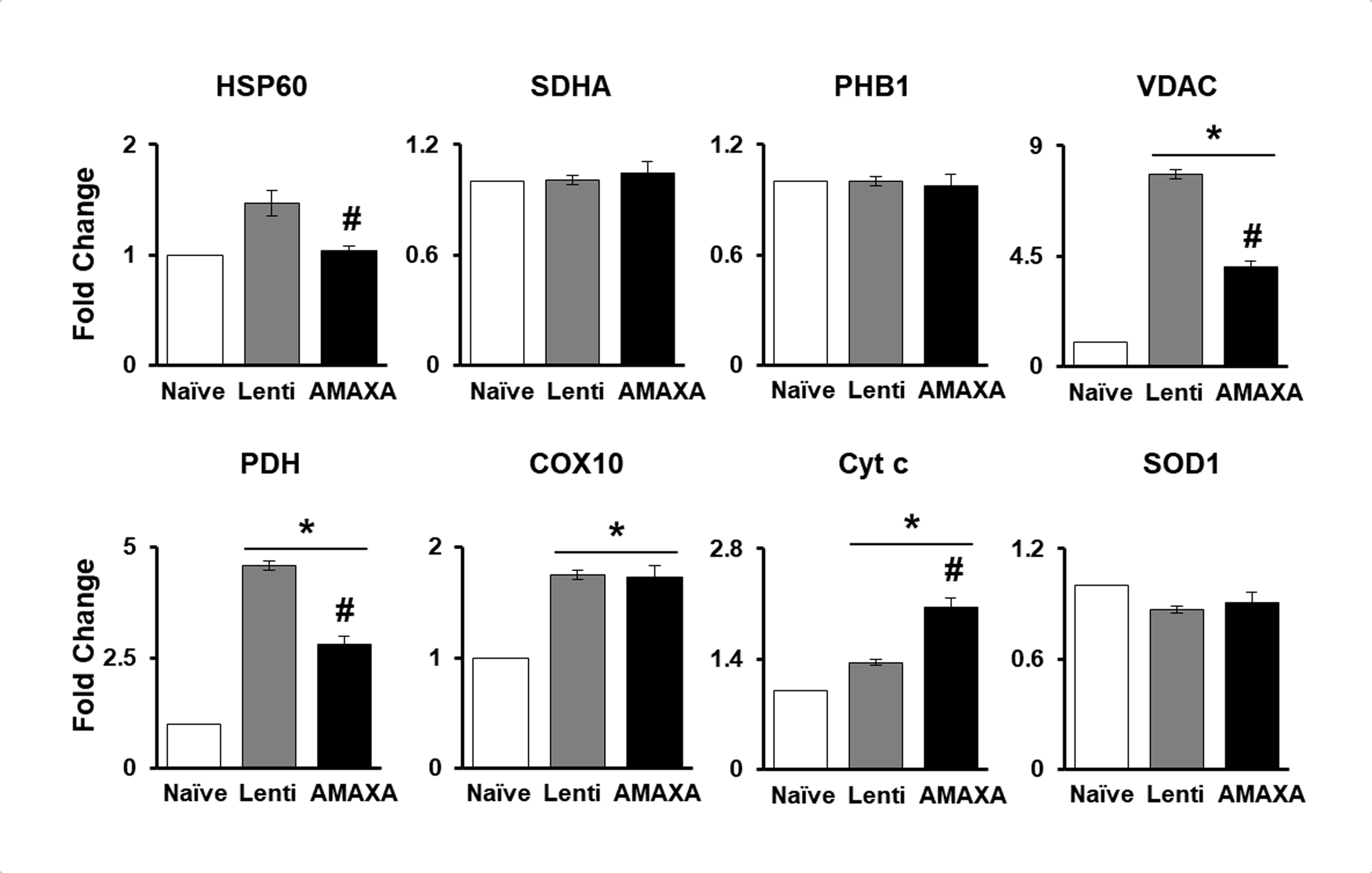

Supplement: Supplementary file 1 — Additional file 1: Supplementary Fig. 1 Quantification of mitochondrial metabolism-specific genes in naïve and PD-MSCsPRL-1 using both gene delivery systems. Data from each group are shown as the mean ± SD. *p < 0.05 versus naïve; #p < 0.05 versus lenti. COX10; cytochrome c oxidase 10, Cyt c; cytochrome c, GAPDH; glyceraldehyde 3-phosphate dehydrogenase, HSP60; heat shock protein 60, PDH; pyruvate dehydrogenase, PHB1; prohibitin 1, SDHA; succinate dehydrogenase, SOD1; superoxide dismutase 1, VDAC; voltage-dependent anion channel. [file 13287_2020_2029_MOESM1_ESM.tif]
